# Baseline and dynamic neutrophil-to-lymphocyte ratio predicts overall survival in unresectable pancreatic cancer following dose-escalated SBRT: a ten-year longitudinal study

**DOI:** 10.3389/fmolb.2026.1877894

**Published:** 2026-06-22

**Authors:** Yupeng Di, Zhijia Sun, Zhuo Song, Lingling Meng

**Affiliations:** 1 Department of Radiation Oncology, Air Force Medical Center, PLA, Beijing, China; 2 Department of Radiation Protection Medicine, School of Preventive Medicine, Fourth Military Medical University, Xi’an, China; 3 Department of Radiation Oncology, Peking University Shougang Hospital, Beijing, China; 4 Department of Radiation Oncology, Senior Department of Oncology, The First Medical Center of PLA General Hospital, Beijing, China

**Keywords:** neutrophil-to-lymphocyte ratio (NLR), pancreatic cancer, precision oncology, prognostic biomarkers, stereotactic body radiation therapy (SBRT), systemic inflammation, treatment-related toxicity

## Abstract

**Background:**

Systemic inflammation is closely associated with progression and therapeutic resistance in pancreatic ductal adenocarcinoma (PDAC). The neutrophil-to-lymphocyte ratio (NLR) serves as a surrogate for the balance between pro-tumor inflammation and adaptive anti-tumor immunity. This study evaluated whether baseline NLR and early post-treatment NLR dynamics were associated with overall survival (OS) in patients with unresectable or medically inoperable PDAC receiving dose-escalated stereotactic body radiation therapy (SBRT).

**Methods:**

We retrospectively analyzed 68 patients with unresectable or medically inoperable PDAC treated with dose-escalated SBRT between January 2013 and December 2023. Baseline NLR was measured within 14 days prior to SBRT, and post-treatment NLR was reassessed approximately 4 weeks after SBRT. The optimal NLR threshold was determined using receiver operating characteristic (ROC) curve analysis for 12-month mortality and was interpreted as an exploratory, cohort-derived cut-off. Survival was estimated using the Kaplan-Meier method, Cox proportional hazards models, and restricted cubic splines (RCS). Clinical utility was **assessed** via decision curve analysis (DCA).

**Results:**

An ROC-derived NLR threshold of 2.96 was identified, demonstrating high discriminatory power with an AUC of 0.872 (p < 0.001). Patients in the NLR-low group (n = 39) achieved a significantly longer median OS compared to the NLR-high group (n = 29) (21.0 vs. 9.0 months, p < 0.0001). In multivariable analysis, NLR < 2.96 remained independently associated with improved OS (Hazard Ratio [HR] = 0.12; 95% CI: 0.06–0.27, p < 0.001) after adjusting for ECOG performance status, M stage, chemotherapy, tumor diameter, albumin, CA19-9, and BED10. Dynamic monitoring showed that patients with “Stably Low” or “Improved” NLR status post-SBRT had significantly better outcomes (p = 0.00084). Joint stratification with CA19-9 further enhanced risk groups, and DCA suggested improved net clinical benefit after incorporating NLR into risk models. No grade 4 or 5 treatment-related toxicities were observed.

**Conclusion:**

Pre-treatment and dynamic NLR may serve as accessible immune-inflammatory biomarkers for patients with unresectable or medically inoperable PDAC undergoing dose-escalated SBRT. The threshold of 2.96 should be regarded as an exploratory cohort-derived value rather than a definitive clinical cut-off, and external validation is required before routine clinical implementation.

## 1 Introduction

Pancreatic ductal adenocarcinoma (PDAC) remains a global oncological challenge and a leading cause of cancer-related mortality, characterized by aggressive local invasion, early systemic dissemination, and resistance to conventional therapies (Grossberg et al., 2020; Di et al., 2025). Despite the clinical integration of systemic treatment strategies, including multi-agent chemotherapy regimens, survival outcomes remain unsatisfactory for many patients with advanced or unresectable disease (Di et al., 2024; Conroy et al., 2011). For patients with locally advanced unresectable, medically inoperable, or selected metastatic PDAC, systemic chemotherapy remains the cornerstone of treatment, while stereotactic body radiation therapy (SBRT) may provide an additional local treatment option for improving local control and symptom management in appropriately selected patients (Von Hoff et al., 2013; Timmerman et al., 2007). However, clinical responses after SBRT remain heterogeneous, and conventional anatomical staging alone may not fully capture the biological variability that influences survival (Petrelli et al., 2017; Herman et al., 2015a). Therefore, accessible biomarkers that reflect both tumor burden and host biological status are needed to improve risk stratification in this population.

The host’s systemic inflammatory status is increasingly recognized as an important determinant of tumor progression and therapeutic resistance. PDAC is typically described as an “immunologically cold” tumor, characterized by dense desmoplastic stroma, impaired immune cell infiltration, and a tumor microenvironment that limits effective anti-tumor immunity (Mantovani et al., 2008; Quail and Joyce, 2013). This unique microenvironment is further shaped by the recruitment and activation of tumor-associated neutrophils (TANs) and myeloid-derived suppressor cells (MDSCs) (Grivennikov et al., 2010; Hanahan and Weinberg, 2011). These myeloid populations can promote tumor growth, angiogenesis, immune evasion, and suppression of cytotoxic lymphocyte activity (Shaul and Fridlender, 2019; Masucci et al., 2019). As a result, systemic inflammatory markers may provide clinically useful information beyond standard radiographic and pathological assessments.

The neutrophil-to-lymphocyte ratio (NLR), a simple and reproducible metric derived from routine peripheral blood counts, provides a quantifiable surrogate of the systemic immune-inflammatory balance (Buonacera et al., 2022; Zahorec, 2021). Elevated NLR values generally reflect a predominance of pro-tumor inflammatory activity and relative impairment of adaptive anti-tumor immune surveillance (Xiong et al., 2021; Templeton et al., 2014). While the prognostic role of baseline NLR has been reported in patients with pancreatic cancer treated with surgery or systemic therapy (An et al., 2010; Aliustaoglu et al., 2010), its clinical relevance in patients receiving dose-escalated SBRT remains less clearly defined. This is particularly relevant because SBRT may influence systemic immune status through local tumor ablation, radiation-induced inflammatory changes, and relative sparing of circulating lymphocytes compared with larger-field conventional radiotherapy (Wild et al., 2016a; Kishi et al., 2016). Furthermore, static baseline measurements provide only a single time-point assessment and may not capture early post-treatment immune-inflammatory changes (Lee et al., 2017; Chen et al., 2018b). Accordingly, the present study aimed to evaluate the association of baseline NLR and early post-SBRT NLR dynamics with overall survival in a retrospective cohort of patients with unresectable or medically inoperable PDAC treated with dose-escalated SBRT.

## 2 Materials and methods

### 2.1 Study design and patient selection

This retrospective single-center cohort study included patients with pancreatic ductal adenocarcinoma (PDAC) who underwent dose-escalated stereotactic body radiation therapy (SBRT) at our institution between January 2013 and December 2023. The institutional database was reviewed to identify patients with surgically unresectable, medically inoperable, locally advanced, or metastatic PDAC who received SBRT as part of their treatment strategy.

A total of 249 patients were initially assessed for eligibility. The inclusion criteria were as follows: 1) pathologically confirmed or clinically/radiographically diagnosed PDAC after multidisciplinary review; 2) disease considered surgically unresectable or medically unsuitable for surgery at the time of SBRT; 3) treatment with SBRT using a dose-escalated regimen with BED10 of at least 60 Gy; 4) available complete blood count within 14 days before SBRT; 5) available post-treatment complete blood count approximately 4 weeks after SBRT; and 6) available follow-up information for survival assessment.

**FIGURE 1:**
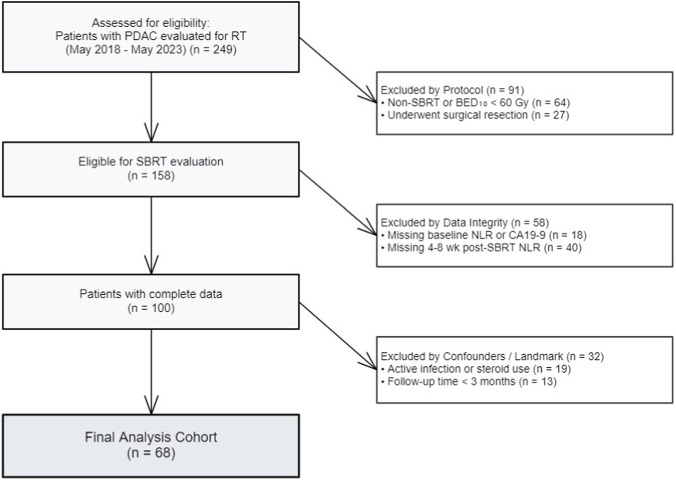
Patient selection flowchart. Flowchart illustrating the screening, exclusion, and final inclusion process for patients with unresectable or medically inoperable pancreatic ductal adenocarcinoma treated with dose-escalated stereotactic body radiation therapy. A total of 249 patients were initially assessed, and 68 patients met all eligibility criteria and were included in the final analysis. SBRT, stereotactic body radiation therapy; BED10, biologically effective dose calculated with an alpha/beta ratio of 10 Gy; NLR, neutrophil-to-lymphocyte ratio.

Patients were excluded if they received non-SBRT radiotherapy, underwent surgery after radiotherapy, had incomplete longitudinal laboratory data, had active infection or uncontrolled inflammatory disease around the time of blood sampling, received systemic corticosteroids during the NLR assessment window, or had insufficient follow-up of less than 3 months without a documented death event. After applying these criteria, 68 patients were included in the final analytic cohort. The detailed screening process and reasons for exclusion are shown in Figure 1.

### 2.2 Staging and definition of unresectability

Clinical staging was reviewed according to the American Joint Committee on Cancer 8th edition staging system. M1 disease was defined as radiographic or pathological evidence of distant metastasis, and AJCC stage IV corresponded directly to M1 disease. Patients without distant metastasis were classified as M0. Therefore, the number of patients with AJCC stage IV disease was identical to the number of patients with M1 disease.

Patients with non-metastatic disease were considered unresectable or medically inoperable based on multidisciplinary evaluation. Locally advanced unresectable disease was generally defined by major vascular involvement or other anatomical factors precluding safe surgical resection. Patients with AJCC stage I/II disease were included only when they were considered medically unsuitable for surgery or technically unresectable after multidisciplinary assessment. This staging review was performed to ensure consistency between metastatic status and AJCC stage classification.

### 2.3 NLR assessment and dynamic classification

Baseline peripheral blood parameters were obtained from routine complete blood count tests performed within 14 days before the initiation of SBRT. NLR was calculated as the absolute neutrophil count divided by the absolute lymphocyte count. Post-treatment NLR was reassessed approximately 4 weeks after completion of SBRT. Paired baseline and post-treatment NLR values were available for all 68 patients included in the final analytic cohort.

The optimal baseline NLR threshold was determined using receiver operating characteristic curve analysis for 12-month mortality, with the cut-off selected by maximizing the Youden index. Because this threshold was derived from the same cohort in which survival outcomes were evaluated, it was regarded as an exploratory, cohort-specific cut-off rather than a validated clinical threshold. To reduce overinterpretation of a single dichotomized value, NLR was also evaluated as a continuous variable using restricted cubic spline analysis.

For dynamic NLR assessment, patients were classified according to their baseline and post-treatment NLR status. “Stably Low” was defined as NLR remaining below the threshold both before and after SBRT. “Improved” was defined as high baseline NLR decreasing to below the threshold after SBRT. “High/Worsened” was defined as persistently high NLR or an increase to high NLR after SBRT. Because post-treatment NLR required survival and laboratory testing at the post-SBRT assessment time point, the dynamic analysis was interpreted as an exploratory landmark analysis based on the approximately 4-week post-SBRT blood test.

### 2.4 SBRT treatment

SBRT was delivered using advanced image-guided radiation platforms. When available and clinically appropriate, MRI-guided adaptive radiotherapy was used to allow daily online plan adaptation. Treatment planning was individualized according to tumor location, tumor size, proximity to organs at risk, and patient-specific anatomy.

Gross tumor volume was delineated using contrast-enhanced computed tomography, magnetic resonance imaging, positron emission tomography when available, and clinical information. Planning target volume margins were determined according to institutional practice, respiratory motion management, image guidance, and the use of adaptive radiotherapy. Dose-escalated regimens were prioritized when organ-at-risk constraints could be safely achieved.

BED10 was recorded for all patients. Organs at risk, including the stomach, duodenum, small bowel, liver, kidneys, and spinal cord, were contoured and constrained according to institutional standards. Particular attention was given to gastrointestinal dose constraints because of the anatomical proximity of pancreatic tumors to the stomach and duodenum. Adaptive planning was used when appropriate to optimize target coverage while minimizing radiation exposure to adjacent organs at risk.

### 2.5 Chemotherapy assessment

Chemotherapy information was manually abstracted from electronic medical records, including whether systemic chemotherapy was administered, the timing of chemotherapy relative to SBRT, and the documented regimen. Chemotherapy timing was categorized as induction chemotherapy before SBRT, concurrent chemotherapy during SBRT, or adjuvant chemotherapy after SBRT. Detailed chemotherapy status, timing, and regimens are summarized in Supplementary Table 1.

Because this retrospective cohort covered a 10-year period, the number of chemotherapy cycles, treatment lines, dose reductions, treatment interruptions, and contraindications to chemotherapy were not consistently documented for all patients. Therefore, chemotherapy was included in the multivariable Cox regression model as a documented yes/no variable. Available details regarding chemotherapy timing and regimen were summarized descriptively.

### 2.6 Follow-up and toxicity assessment

Patients were generally followed 4–6 weeks after completion of SBRT and then every 2–3 months during the first 2 years. Longer follow-up intervals were used thereafter according to disease status, treatment response, and clinical condition. Follow-up evaluations included physical examination, complete blood count, liver and renal function tests, CA19-9 assessment when available, and abdominal imaging using computed tomography or magnetic resonance imaging. Survival status was determined from medical records, follow-up visits, and telephone follow-up when necessary.

Treatment-related toxicity was evaluated according to the Common Terminology Criteria for Adverse Events, version 5.0. Acute toxicity was defined as adverse events occurring within 90 days after initiation of SBRT, whereas late toxicity was defined as adverse events occurring more than 90 days after completion of SBRT. Toxicity information was abstracted from clinical notes, laboratory reports, imaging records, emergency visits, and hospitalization records. Treatment-related toxicities are summarized in Supplementary Table 2.

### 2.7 Missing data and statistical analysis

Patients with missing baseline NLR, missing post-treatment NLR, incomplete survival data, or insufficient follow-up were excluded during cohort selection, as shown in Figure 1. No statistical imputation was performed because of the small sample size and retrospective study design. Complete-case analysis was used for all survival models.

Overall survival was defined as the interval from the first day of SBRT to death from any cause or last follow-up. For exploratory dynamic NLR analysis, survival was interpreted using a landmark framework based on the post-SBRT NLR assessment time point. Survival distributions were estimated using the Kaplan-Meier method and compared using the log-rank test. Numbers at risk were displayed below Kaplan-Meier curves.

Univariate and multivariable Cox proportional hazards regression models were used to evaluate associations between clinical variables and OS. The multivariable model included NLR, ECOG performance status, M stage, chemotherapy status, tumor diameter, albumin, CA19-9, and BED10. T stage, N stage, and AJCC stage were not included in the multivariable model to reduce multicollinearity with tumor extent and metastatic status.

Restricted cubic spline analysis was performed to evaluate the potential non-linear association between continuous baseline NLR and mortality risk. Decision curve analysis was used to estimate the potential clinical net benefit of incorporating NLR into a risk prediction model at the 12-month time point. All statistical tests were two-sided, and p < 0.05 was considered statistically significant.

## 3 Results

### 3.1 Study population and selection process

A total of 249 patients with unresectable or medically inoperable PDAC were initially screened. As shown in the standardized patient selection flowchart (Figure 1), 181 patients were excluded because of radiotherapy protocol non-compliance, incomplete longitudinal laboratory data, clinical confounders, or insufficient follow-up. Ultimately, 68 patients met all eligibility criteria and were included in the final analysis.

Among the final cohort, 57 patients were classified as M0 and 11 patients as M1. Accordingly, AJCC stage IV disease was present in 11 patients, corresponding directly to the M1 population. Patients with AJCC stage I/II disease were included only if they were considered medically inoperable or technically unsuitable for surgical resection after multidisciplinary evaluation. Baseline clinicopathological characteristics stratified by NLR group are summarized in Table 1.

**TABLE 1:** Baseline patient characteristics stratified by NLR group.

Variables	All patients (n = 68)	NLR-high (n = 29)	NLR-low (n = 39)	*p*-value
Age, n (%)				1.000
<65	37 (54.4)	16 (55.2)	21 (53.8)	
≥65	31 (45.6)	13 (44.8)	18 (46.2)	
Gender, n (%)				0.621
Male	39 (57.4)	18 (62.1)	21 (53.8)	
Female	29 (42.6)	11 (37.9)	18 (46.2)	
ECOG PS, n (%)				0.042
0	16 (23.5)	3 (10.3)	13 (33.3)	
1	52 (76.5)	26 (89.7)	26 (66.7)	
Primary site, n (%)				0.436
Head	48 (70.6)	22 (75.9)	26 (66.7)	
Body/Tail	20 (29.4)	7 (24.1)	13 (33.3)	
T stage, n (%)				0.454
T2	2 (2.9)	1 (3.4)	1 (2.6)	
T3	20 (29.4)	6 (20.7)	14 (35.9)	
T4	46 (67.6)	22 (75.9)	24 (61.5)	
N stage, n (%)				0.802
N0	45 (66.2)	18 (62.1)	27 (69.2)	
N1	21 (30.9)	10 (34.5)	11 (28.2)	
N2	2 (2.9)	1 (3.4)	1 (2.6)	
M stage, n (%)				0.184
M0	57 (83.8)	22 (75.9)	35 (89.7)	
M1	11 (16.2)	7 (24.1)	4 (10.3)	
AJCC 8th stage, n (%)				0.133
I/II	16 (23.5)	4 (13.8)	12 (30.8)	
III	41 (60.3)	18 (62.1)	23 (59.0)	
IV	11 (16.2)	7 (24.1)	4 (10.3)	
Tumor diameter	4.00 [3.00, 5.00]	4.00 [3.00, 5.00]	4.00 [3.10, 5.00]	0.708
Albumin	43.22 (5.55)	44.54 (7.88)	42.33 (2.95)	0.124
CA19-9	293.60 [74.06, 759.09]	295.20 [170.30, 855.36]	263.32 [33.08, 698.35]	0.191
Received chemotherapy, n (%)				1.000
Yes	24 (35.3)	10 (34.5)	14 (35.9)	
No	44 (64.7)	19 (65.5)	25 (64.1)	
BED10	94.50 [78.00, 102.70]	89.60 [81.00, 94.50]	94.50 [78.00, 102.70]	0.241
Biliary intervention, n (%)				0.799
Yes	24 (35.3)	11 (37.9)	13 (33.3)	
No	44 (64.7)	18 (62.1)	26 (66.7)	

Abbreviations: NLR: neutrophil-to-lymphocyte ratio; BED10: biologically effective dose; ECOG PS: Eastern Cooperative Oncology Group PS.

Data presented as n (%), mean (SD), or median [IQR]. AJCC, Stage I/II, patients were deemed medically or technically unresectable by multidisciplinary consensus. Biliary intervention includes stenting or drainage.

### 3.2 NLR threshold and baseline stratification

Comparative analysis between the NLR-high and NLR-low groups showed that most baseline variables, including age, sex, primary tumor site, T stage, N stage, M stage, AJCC stage, tumor diameter, albumin, CA19-9, BED10, chemotherapy status, and biliary intervention, were not significantly different between groups (Table 1). However, ECOG performance status differed between groups, with patients in the NLR-low group more frequently having ECOG PS 0 (p = 0.042).

**FIGURE 2:**
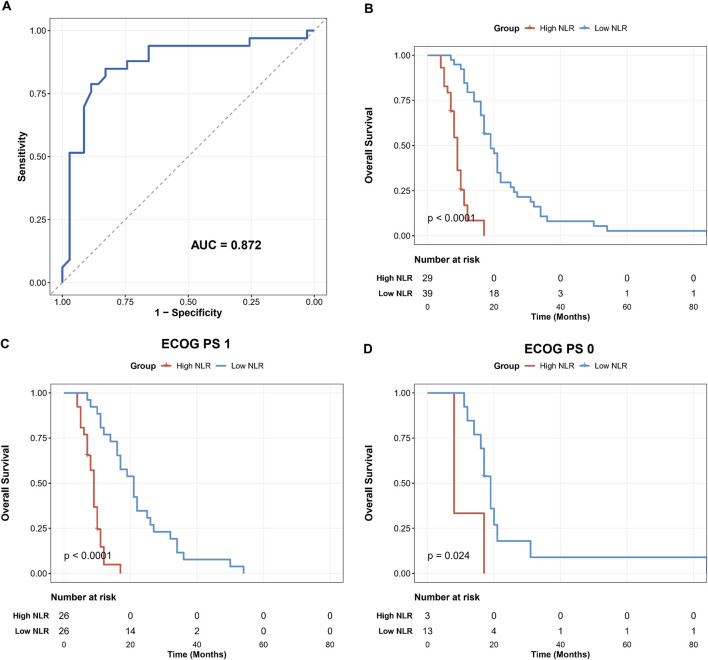
Prognostic performance of baseline NLR and survival stratification. **(A)** Receiver operating characteristic curve analysis of baseline neutrophil-to-lymphocyte ratio for predicting 12-month mortality, showing an area under the curve of 0.872. The optimal ROC-derived NLR threshold was 2.96. **(B)** Kaplan-Meier survival curves for the entire cohort stratified by baseline NLR status. Patients with NLR < 2.96 had significantly longer overall survival than those with NLR ≥ 2.96. **(C)** Kaplan-Meier survival curves among patients with ECOG performance status 1. **(D)** Kaplan-Meier survival curves among patients with ECOG performance status 0. Numbers at risk are shown below the Kaplan-Meier plots. NLR, neutrophil-to-lymphocyte ratio; ROC, receiver operating characteristic; AUC, area under the curve; OS, overall survival; ECOG PS, Eastern Cooperative Oncology Group performance status.

ROC curve analysis for 12-month mortality **identified** an NLR threshold of 2.96, with an AUC of 0.872 (Figure 2A). Patients were subsequently stratified into the NLR-low group (n = 39) and NLR-high group (n = 29). Patients in the NLR-low group had significantly longer median OS than those in the NLR-high group (21.0 vs. 9.0 months, p < 0.0001; Figure 2B). The prognostic separation remained evident in subgroup analyses of patients with ECOG PS 1 (Figure 2C) and ECOG PS 0 (Figure 2D). Numbers at risk are displayed below the Kaplan-Meier curves.

### 3.3 Independent survival predictors and morphological distribution

The results of univariate and multivariable Cox proportional hazards regression analyses are shown in Table 2. In univariate analysis, baseline NLR, M stage, AJCC stage, and BED10 were associated with OS. In the multivariable model adjusted for NLR, ECOG performance status, M stage, chemotherapy, tumor diameter, albumin, CA19-9, and BED10, NLR < 2.96 remained independently associated with improved OS (HR = 0.12; 95% CI: 0.06–0.27; p < 0.001). M1 disease was independently associated with worse OS (HR = 17.77; 95% CI: 3.74–84.46; p < 0.001). Chemotherapy status was not significantly associated with OS in this model (HR = 0.78; 95% CI: 0.36–1.71; p = 0.537).

**TABLE 2:** Cox regression analysis of overall survival predictors.

Variable	Univariate HR (95% CI)	P-value	Multivariate HR (95% CI)	P-value
NLR				
≥2.96	Ref		Ref	
<2.96	0.12 (0.06–0.23)	<0.001	0.12 (0.06–0.27)	<0.001
Age				
≥65	Ref		-	-
<65	0.84 (0.51–1.39)	0.507	-	-
Gender				
Male	Ref		-	-
Female	0.81 (0.49–1.34)	0.409	-	-
ECOG PS				
0	Ref		Ref	
1	1.40 (0.77–2.56)	0.266	0.93 (0.45–1.92)	0.848
Primary site				
Head	Ref		-	-
Body/Tail	0.75 (0.43–1.30)	0.300	-	-
T Stage				
T2/T3	Ref		-	-
T4	1.19 (0.70–2.02)	0.524	-	-
N stage				
N0	Ref		-	-
N1/N2	1.12 (0.66–1.90)	0.669	-	-
M Stage				
M0	Ref		Ref	
M1	2.76 (1.42–5.36)	0.003	17.77 (3.74–84.46)	<0.001
AJCC 8th stage				
Stage I/II	Ref		-	-
Stage III	1.30 (0.71–2.41)	0.397	-	-
Stage IV	3.33 (1.49–7.45)	0.003	-	-
Received chemotherapy				
No	Ref		Ref	
Yes	0.95 (0.53–1.68)	0.852	0.78 (0.36–1.71)	0.537
Tumor diameter	0.88 (0.70–1.11)	0.293	0.94 (0.72–1.22)	0.636
Albumin	1.02 (0.96–1.09)	0.485	1.03 (0.97–1.08)	0.376
CA19-9	1.00 (1.00–1.00)	0.124	1.00 (1.00–1.00)	0.162
BED10	0.98 (0.96–1.00)	0.046	0.99 (0.96–1.01)	0.269
Biliary intervention				
No	Ref		-	-
Yes	1.12 (0.67–1.86)	0.673	-	-

The multivariate Cox model was adjusted for NLR, ECOG PS, M stage, chemotherapy, tumor diameter, albumin, CA19-9, and BED10. T/N classifications and AJCC, staging were excluded from the multivariate analysis to prevent multicollinearity.

**FIGURE 3:**
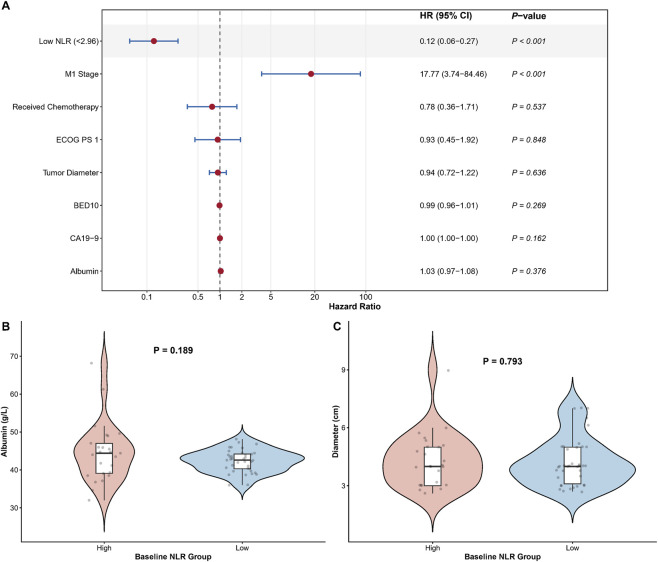
Multivariable predictors of overall survival and morphological correlates. **(A)** Forest plot showing the multivariable Cox proportional hazards regression analysis for overall survival. NLR < 2.96 was independently associated with improved overall survival, whereas M1 disease was associated with worse overall survival after adjustment for clinically relevant covariates. **(B)** Violin plot comparing serum albumin levels between the NLR-high and NLR-low groups. **(C)** Violin plot comparing primary tumor diameter between the NLR-high and NLR-low groups. NLR, neutrophil-to-lymphocyte ratio; HR, hazard ratio; CI, confidence interval; OS, overall survival; CA19-9, carbohydrate antigen 19-9; BED10, biologically effective dose calculated with an alpha/beta ratio of 10 Gy.

Although baseline NLR was strongly associated with survival, violin plot analyses showed no significant difference in albumin levels between the NLR-high and NLR-low groups (p = 0.124; Figure 3B) or in primary tumor diameter between groups (p = 0.708; Figure 3C). These findings suggest that NLR may capture a host inflammatory dimension that is not fully reflected by nutritional status or primary tumor size alone.

### 3.4 Dynamic NLR monitoring and joint risk models

**FIGURE 4:**
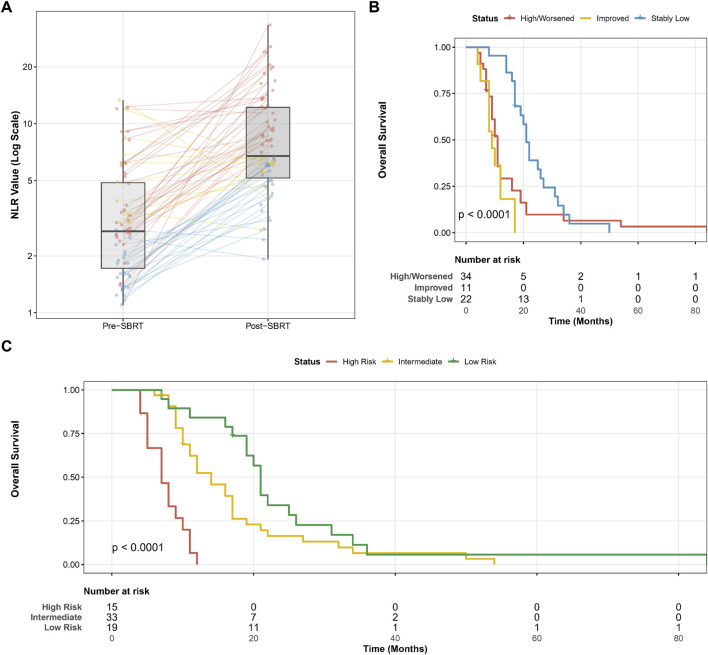
Dynamic NLR monitoring and integrated risk stratification. **(A)** Spaghetti plot showing individual changes in NLR from baseline to approximately 4 weeks after SBRT. **(B)** Kaplan-Meier survival curves according to dynamic NLR transition status: Stably Low, Improved, and High/Worsened. Patients with Stably Low or Improved NLR status had better overall survival than those with High/Worsened status in exploratory landmark analysis. **(C)** Kaplan-Meier survival curves based on joint stratification by baseline NLR and CA19-9 status. Patients were classified as Low Risk when both biomarkers were low, Intermediate Risk when one biomarker was high, and High Risk when both biomarkers were high. Numbers at risk are shown below the Kaplan-Meier plots where applicable. NLR, neutrophil-to-lymphocyte ratio; SBRT, stereotactic body radiation therapy; CA19-9, carbohydrate antigen 19-9; OS, overall survival.

Paired baseline and post-treatment NLR values were available for all 68 patients in the final cohort. Longitudinal tracking demonstrated substantial interpatient heterogeneity in systemic inflammatory changes after SBRT (Figure 4A). In exploratory landmark analysis based on the approximately 4-week post-SBRT assessment, patients with “Stably Low” or “Improved” NLR status had significantly better OS than those with “High/Worsened” status (p = 0.00084; Figure 4B). This finding suggests that early post-treatment inflammatory remodeling may provide additional prognostic information beyond the baseline NLR value.

The integration of baseline NLR and CA19-9 further stratified patients into three risk groups: Low Risk, defined as both biomarkers low; Intermediate Risk, defined as one biomarker high; and High Risk, defined as both biomarkers high. These groups demonstrated markedly different survival trajectories (p < 0.0001; Figure 4C), supporting the potential value of combining host inflammatory status with tumor-related biomarkers.

### 3.5 Non-linear association and clinical utility

**FIGURE 5:**
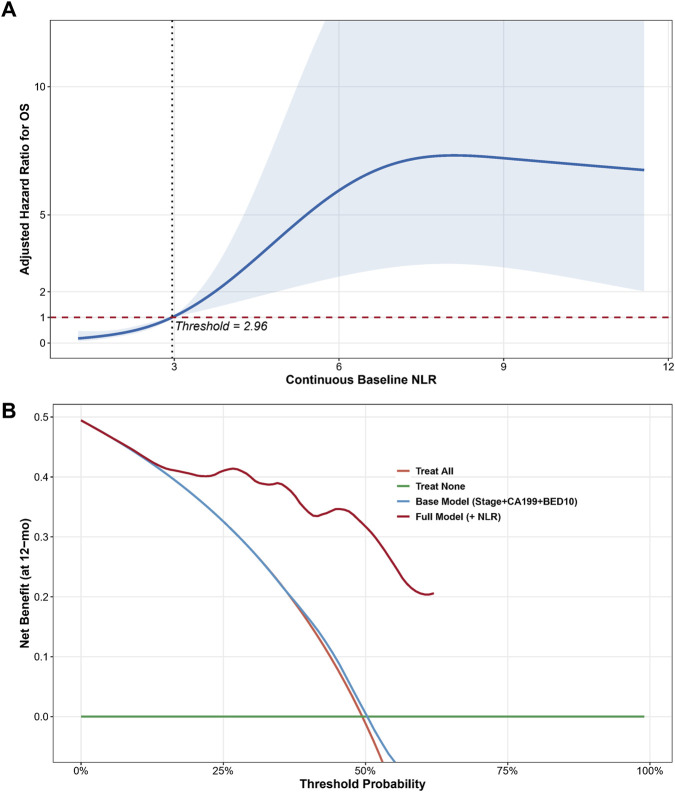
Non-linear risk modeling and clinical decision analysis. **(A)** Restricted cubic spline analysis showing the non-linear association between continuous baseline NLR and the estimated hazard of death. The vertical dashed line indicates the ROC-derived threshold of 2.96. **(B)** Decision curve analysis evaluating the net clinical benefit of different prediction models for 12-month mortality. The full model incorporating NLR, M stage, and CA19-9 showed greater net benefit than the base model including M stage and CA19-9 alone across a range of threshold probabilities. NLR, neutrophil-to-lymphocyte ratio; ROC, receiver operating characteristic; RCS, restricted cubic spline; DCA, decision curve analysis; CA19-9, carbohydrate antigen 19-9.

Restricted cubic spline analysis demonstrated a non-linear association between continuous baseline NLR and mortality risk (Figure 5A). The estimated risk increased as NLR rose, with a more apparent upward trend around the ROC-derived threshold of 2.96. Because the threshold was derived from this cohort, this finding should be interpreted as supportive rather than confirmatory.

Decision curve analysis **suggested** that incorporating NLR into the risk model provided a higher net clinical benefit than the base model including M stage and CA19-9 alone across a range of clinically relevant threshold probabilities (Figure 5B). These results indicate that NLR may add clinically useful prognostic information, although external validation is required before routine clinical implementation.

## 4 Discussion

This retrospective 10-year cohort study suggests that baseline NLR and early post-SBRT NLR dynamics are associated with overall survival in patients with unresectable or medically inoperable PDAC treated with dose-escalated SBRT. Patients with baseline NLR below the ROC-derived threshold of 2.96 had significantly longer survival than those with elevated NLR. This association remained significant after adjustment for clinically relevant covariates, including ECOG performance status, M stage, chemotherapy status, tumor diameter, albumin, CA19-9, and BED10. However, because the threshold was derived from the same cohort in which survival outcomes were evaluated, it should be interpreted as an exploratory, cohort-specific cut-off rather than a definitive clinical threshold.

The biological rationale for the association between elevated NLR and worse prognosis is supported by the established role of systemic inflammation in tumor progression and immune suppression. Neutrophil expansion may reflect activation of pro-tumor myeloid pathways, including myeloid-derived suppressor cells and tumor-associated neutrophils, which can promote angiogenesis, tissue invasion, metastatic niche formation, and suppression of cytotoxic lymphocyte function (Gabrilovich and Nagaraj, 2009; Talmadge and Gabrilovich, 2013). Neutrophils may further contribute to metastatic progression through inflammatory cytokines, reactive oxygen species, and immunosuppressive mediators (Coffelt et al., 2015; Ocana et al., 2017). In contrast, lymphocytes are central mediators of adaptive anti-tumor immunity. Therefore, an elevated NLR may reflect both enhanced innate inflammatory activity and impaired adaptive immune surveillance, which together may contribute to inferior outcomes. Nevertheless, these biological explanations should be regarded as mechanistic hypotheses rather than direct evidence from the present cohort, because this study did not include tumor microenvironment profiling, immune-cell phenotyping, cytokine assays, or functional immune analyses.

The prognostic relevance of NLR may be particularly important in the setting of SBRT. Compared with conventional large-field radiotherapy, SBRT may reduce unnecessary exposure of circulating lymphocytes and uninvolved lymphoid tissues, which may help preserve systemic immune competence (Wild et al., 2016b; Fukunaga et al., 2004). In our cohort, NLR retained prognostic value even among patients with preserved performance status, suggesting that systemic inflammatory markers may capture biological vulnerability not fully reflected by ECOG performance status or conventional anatomical staging (Feliciano et al., 2017). Previous studies have similarly reported that inflammation-based biomarkers, including NLR, are associated with outcomes in pancreatic cancer across different treatment settings (Stotz et al., 2013; Martin et al., 2014; Yang et al., 2015). Nevertheless, the present findings should be regarded as associative rather than causal, given the retrospective design and the potential for residual confounding.

The dynamic NLR analysis provides additional prognostic information but should be interpreted with particular caution. Patients whose NLR remained low or improved after SBRT had better survival than those with persistently elevated or worsened NLR. This pattern may reflect reduced tumor-driven inflammatory signaling, recovery of host immune balance, or differences in overall treatment responsiveness (Luo et al., 2019; Kim et al., 2019; Chen et al., 2018a). However, post-treatment NLR was assessed approximately 4 weeks after SBRT, and patients had to survive and undergo laboratory testing at that time to be included in the dynamic assessment. Although we interpreted this component as an exploratory landmark analysis, residual landmark time bias and selection bias cannot be fully excluded. Therefore, dynamic NLR should be considered hypothesis-generating and requires prospective validation using predefined post-treatment sampling time points. Importantly, the present study does not establish that NLR dynamics can be used as a real-time monitor of therapeutic efficacy or immune reactivation. Such an application would require prospective validation with serial biomarker sampling, standardized systemic therapy, and predefined clinical decision rules.

Systemic chemotherapy is a major determinant of survival in locally advanced and metastatic PDAC and may also influence peripheral immune-inflammatory biomarkers. In the present study, chemotherapy status, timing, and regimens were summarized in Supplementary Table 1, and chemotherapy was included as a covariate in the multivariable Cox model. However, detailed information regarding chemotherapy cycles, treatment lines, dose intensity, treatment interruptions, and contraindications was not consistently available because of the retrospective nature of the cohort. This limitation restricts our ability to fully account for the impact of systemic therapy. Patients with persistently elevated NLR or combined elevation of NLR and CA19-9 may represent a high-risk subgroup requiring more intensive systemic treatment, closer surveillance, or enrollment in clinical trials rather than reliance on local therapy alone (Suker et al., 2016; Herman et al., 2015b; Reyngold et al., 2019). However, our data should not be interpreted as evidence that NLR can determine the selection, timing, or intensity of systemic therapy.

The integration of NLR with CA19-9 may offer a more comprehensive risk stratification approach. CA19-9 primarily reflects tumor-associated secretory activity and tumor burden, whereas NLR may reflect host inflammatory and immune status (Wong et al., 2014; Inoue et al., 2015). In this study, patients with both elevated NLR and elevated CA19-9 had the poorest outcomes, suggesting that combined tumor-related and host-related biomarkers may identify a particularly high-risk phenotype. Decision curve analysis also suggested that adding NLR to a model based on M stage and CA19-9 may improve net clinical benefit (Luo et al., 2017; Gunderson et al., 2018). However, these results should be considered exploratory because the prediction model and decision curve analysis were derived from the same retrospective dataset and have not yet been externally validated. Therefore, the combined NLR–CA19-9 model should currently be viewed as a hypothesis-generating risk-stratification tool rather than a validated decision-making algorithm.

This study has several limitations. First, it was retrospective and single-center in design, with a relatively small sample size of 68 patients. Second, the NLR threshold of 2.96 was derived and tested within the same dataset, creating a risk of overfitting and optimism bias. Although continuous NLR analysis and restricted cubic spline modeling were performed to support the observed association, external validation remains necessary before this threshold can be used in routine clinical practice. Third, chemotherapy was incompletely characterized beyond documented receipt, timing, and regimen, and more granular systemic therapy variables could not be consistently incorporated into the multivariable model. Fourth, dynamic NLR assessment may be affected by landmark time bias because post-treatment NLR required survival and laboratory testing after SBRT. Fifth, although patients with active infection and corticosteroid exposure were excluded, other inflammatory conditions, biliary events, nutritional factors, and occult infections may have influenced NLR values. Finally, the cohort included clinically heterogeneous patients, including medically inoperable non-metastatic patients and selected patients with metastatic disease. Although staging was reviewed according to AJCC eighth edition criteria and AJCC stage IV corresponded directly to M1 disease, residual heterogeneity remains. In addition, this study did not evaluate immunotherapy, immune checkpoint inhibitors, or treatment combinations involving immunomodulatory agents. Therefore, no conclusion can be drawn regarding whether NLR dynamics predict benefit from immune-based strategies or whether they should guide future immunotherapy-SBRT protocols.

Despite these limitations, the present study suggests that NLR may help identify risk groups among patients with unresectable or medically inoperable PDAC receiving dose-escalated SBRT. Future prospective studies should validate the NLR threshold, incorporate standardized chemotherapy documentation, use predefined landmark time points for post-treatment biomarker assessment, and evaluate whether NLR-guided risk stratification adds incremental value beyond established clinical and tumor-related factors. Rather than serving as a real-time therapeutic monitor at this stage, NLR should be considered an accessible exploratory biomarker that may help generate hypotheses for prospective trials. Further studies are needed before NLR dynamics can be used to guide treatment intensification, surveillance frequency, or integration with novel systemic therapies (Topalian et al., 2015; Maitra and Hruban, 2008).

## 5 Conclusion

In this retrospective single-center cohort, baseline NLR and early post-SBRT NLR dynamics were associated with overall survival in patients with unresectable or medically inoperable PDAC treated with dose-escalated SBRT. Patients with NLR below the **cohort-derived** threshold of 2.96 had significantly longer survival, and those whose NLR remained low or improved after SBRT also showed favorable outcomes.

These findings suggest that NLR may serve as a simple, inexpensive, and clinically accessible biomarker reflecting the systemic immune-inflammatory status of the host. However, the identified threshold should be considered exploratory because it was derived from the same cohort used for outcome assessment. Larger prospective studies with standardized chemotherapy documentation, predefined landmark analyses, and external validation are needed before NLR-guided risk stratification can be incorporated into routine clinical decision-making.

## Data Availability

The original contributions presented in the study are included in the article/Supplementary Material, further inquiries can be directed to the corresponding authors.
