# Supplementary material for: Baseline and dynamic neutrophil-to-lymphocyte ratio predicts overall survival in unresectable pancreatic cancer following dose-escalated SBRT: a ten-year longitudinal study
Source: Front Mol Biosci. 2026 Jun 22;13:1877894. doi: 10.3389/fmolb.2026.1877894 (PMC13333483; doi:10.3389/fmolb.2026.1877894)
Supplement: Supplementary file 1 [file Table1.docx]

**Supplementary Table 1. Summary of Chemotherapy Status, Timing, and Regimens (n = 68)**

| **Characteristics** | **Frequency (n = 68)** | **Percentage (%)** |
| --- | --- | --- |
| **Overall Chemotherapy Status** |  |  |
| Received Chemotherapy | 24 | 35.3 |
| SBRT alone / No records | 44 | 64.7 |
| **Timing of Chemotherapy** |  |  |
| Induction (Pre-SBRT) [a] | 15 | 22.1 |
| Concurrent (with SBRT) [b] | 5 | 7.4 |
| Adjuvant (Post-SBRT) [c] | 4 | 5.9 |
| **Specific Regimens** |  |  |
| Gemcitabine-based [d] | 12 | 17.6 |
| S-1-based [e] | 6 | 8.8 |
| Platinum-based (e.g., Oxaliplatin) | 2 | 2.9 |
| Others / Combined regimens | 4 | 5.9 |

***Footnotes****:[a] Induction chemotherapy was defined as systemic therapy initiated more than 14 days before the first fraction of SBRT.*

*[b] Concurrent therapy was defined as chemotherapy administered between the first and last SBRT fractions.*

*[c] Adjuvant chemotherapy was defined as therapy initiated within 3 months following the completion of SBRT.*

*[d] Gemcitabine-based regimens include gemcitabine monotherapy or gemcitabine combined with nab-paclitaxel.*

*[e] S-1-based regimens include S-1 monotherapy or S-1 combined with other agents (e.g., erlotinib).*

*Abbreviations: SBRT, stereotactic body radiation therapy; PDAC, pancreatic ductal adenocarcinoma.*

**Supplementary Table 2. Treatment-Related Toxicities Classified by CTCAE v5.0**

| **Adverse Events** | **Grade 1-2, n (%)** | **Grade 3, n (%)** | **Grade 4-5, n (%)** |
| --- | --- | --- | --- |
| **Acute Toxicity (< 3 months)** [a] |  |  |  |
| Fatigue | 15 (22.1) | 1 (1.5) | 0 (0.0) |
| Nausea / Vomiting | 9 (13.2) | 0 (0.0) | 0 (0.0) |
| Anorexia | 11 (16.2) | 0 (0.0) | 0 (0.0) |
| Leukopenia | 6 (8.8) | 2 (2.9) | 0 (0.0) |
| Abdominal Pain | 4 (5.9) | 0 (0.0) | 0 (0.0) |
| **Late Toxicity (≥ 3 months)** [b] |  |  |  |
| Radiation gastritis / enteritis | 2 (2.9) | 0 (0.0) | 0 (0.0) |
| Gastrointestinal bleeding / Ulcer | 1 (1.5) | 1 (1.5) | 0 (0.0) |
| Biliary obstruction / Re-stenosis | 3 (4.4) | 1 (1.5) | 0 (0.0) |

*Footnotes:[a] Acute toxicities were defined as adverse events related to SBRT occurring within 90 days after the initiation of radiotherapy.*

*[b] Late toxicities were defined as adverse events occurring more than 90 days after the completion of SBRT.*

*Notes: Toxicity grading was performed according to the Common Terminology Criteria for Adverse Events (CTCAE), version 5.0. No treatment-related deaths (Grade 5) occurred within the study cohort.*

*Abbreviations: SBRT, stereotactic body radiation therapy; GI, gastrointestinal.*
